# 

**Published:** 1845-12

**Authors:** 


*To our Subscribers in Great Britain.* In the last number of the Journal, we expressed the hope that we had effected arrangements by which our subscribers in Great Britain, would hereafter be promptly supplied with the Journal, but in consequence of the temporary absence of our agent, some delay in their delivery has occurred. We must, therefore, claim a little indulgence, as the delay is not attributable to any fault of ours. We are now trying to effect a permanent arrangement—one that will prevent for the future, any irregularity or delay in this matter. We are greatly indebted to the profession in Great Britain for the liberal manner in which they have patronized the Journal from its commencement, and we sincerely regret that they should have any cause of complaint, and they may rest assured, that we shall do all we can to prevent it. If we succeed in consummating the arrangement which we are now endeavoring to make, all difficulty will, for the future, be prevented.

*To Correspondents.*—Observations on Artificial Obturators and Palates, read before the Mississippi Valley Association of Surgeon Dentists, by B. B. Brown, M. D., D. D. S., was received too late for publication in the present number of the Journal. It shall appear in the next . . . . C. D. S. is referred to Dr. Westcott's article on the use of arsenic, as a dental therapeutic agent, for the information which he asks . . . . Dr. R. will accept our thanks for the information he furnished. We have made his case known to the Recording Secretary of the American Society of Dental Surgeons. We will reply to his letter, so soon as we can find leisure to do so . . . . Dr. C. H. is informed that the Journal is regularly sent to his address, but as he did not receive the first number of 6th volume, we sent another. Dr. C. is also informed, that the Journal is regularly sent to him.—*Balt. Ed.*

*Convention of Dentists.*—A convention of Surgeon Dentists is to be held in Philadelphia, on the 15th of this month, for the purpose of organizing a State Society. We hope it will be well attended, and that this object will be accomplished; believing, if it be composed of well-informed and scientific practitioners, that it will be productive of good, both to themselves and the inhabitants of the state. The advantages of association to dentists, are as great as they are to general surgeons and physicians. By this means, the knowledge of many is made available to each and every one. By coming together at stated periods, the members of the profession in Pennsylvania, or such as shall belong to the society, will have an opportunity of comparing their respective modes of practice, and of making known any new discoveries or improvements which they may make in this branch of the healing art.

We wish those of our professional brethren who are engaged in this matter, success; and, as they have honored us with an invitation to attend the convention, we shall endeavor to be present on the occasion. Dr. Robert Arthur, we understand, is to deliver the opening address.—*Balt. Ed.*

J. Robinson, Esq., D. D. S. of London, having consented to become our correspondent, is accredited as such, and will receive and forward to us any communications for the Journal, with which our brethren in England may favor us. There are many eminent practitioners and able writers among the members of the profession in Great Britain, and we should be happy of the opportunity of placing the names of some of them on the list of our contributors—we hope soon to be able to do so.

